# CPR-Induced Life-Threatening Hemothorax in a Rescue PCI Patient: Case Report and Brief Challenges of Regional Centers

**DOI:** 10.3390/reports7030069

**Published:** 2024-08-12

**Authors:** Vaikunthan Thanabalasingam, Clement Tan, Chaminda Sella Kapu, Mark Daniel Higgins, Zhihua Zhang

**Affiliations:** 1 Coronary Care Unit & Cardiac Catheterisation Laboratory, Department of Cardiology, Mackay Base Hospital, Mackay, QLD 4740, Australia; 2 The Prince Charles Hospital, Brisbane, QLD 4740, Australia; 3 College of Medicine and Dentistry, James Cook University, Townsville, QLD 4740, Australia

**Keywords:** cardiopulmonary resuscitation, hemothorax, hemorrhagic shock, respiratory compromise, intercostal arteries

## Abstract

**Background:** Cardiopulmonary resuscitation (CPR) is performed in cardiac arrests. There exist life support guidelines for individuals in performing effective CPR. CPR-related bleeding and hemothoraces are rare. Intercostal artery rupture leading up to shock and respiratory compromise in such situations is rare. Here, we present a unique case with a management dilemma while discussing challenges and guidance to regional centers. **Case presentation:** A 49-year-old Caucasian male experienced an out-of-hospital cardiac arrest which required bystander cardiopulmonary resuscitation from a colleague prior to commencement of lysis protocol at the local hospital. Transfer was later arranged to the nearest cardiac catheterization laboratory where a rescue percutaneous coronary intervention was performed in the left anterior descending artery that required strict dual antiplatelet use. Beneath the shroud of these events was a life-threatening right-sided hemothorax from rupture of intercostal arteries that occurred during initial resuscitation. Astute recognition of this post-percutaneous coronary intervention resulted in eventual transfer of the patient to a tertiary center where the source and the collection of the bleed was addressed. The patient’s took a great trajectory to improvement. **Conclusions:** A regional center poses many challenges and limitations. Massive bleeding from intercostal arteries leading to hemorrhagic shock and respiratory compromise from an expanding hemothorax post-CPR is rare. Post-percutaneous coronary intervention use of dual antiplatelets posed a management dilemma that prompted assistance from tertiary counterparts. Clinicians should be astute and quick in assessing and providing care.

## 1. Introduction

Cardiopulmonary resuscitation (CPR) is an emergency procedure performed in cardiac arrests. It consists of chest compressions and artificial ventilation to manually preserve circulatory flow and oxygenation of the body’s vital organs [1]. Optimal CPR is achieved through conducting chest compressions between a depth of 5 cm to 6 cm, at an approximate rate of 100 to 120 per minute for 30 times before providing two breaths to conclude one cycle [2,3]. This cycle may then repeat pending the next course of clinical findings. This is guided by basic and advanced life support guidelines. CPR-related bleeding and fractures are not uncommon [4]. Azygos vein rupture, internal mammary artery injuries, coronary artery rupture, gastric artery rupture, and intercostal artery damage have all been previously reported and all carry high mortality risks [2,4,5,6,7]. These reported vessels can lead to a myriad of complications such as hemothoraces, intra-abdominal bleeding, and cardiac tamponade [1,2]. The current incidence of hemothoraces, as a result of CPR, is uncommon at just under 24% [1]. However, hemothoraces can be fatal if not addressed in a timely and effective manner. 

Here, we discuss a case in a regional Australian hospital of a life-threatening unilateral hemothorax post-CPR performed on an out-of-hospital cardiac arrest that warranted immediate rescue percutaneous coronary intervention (PCI) on arrival. This was directly followed by a management dilemma that eventually required a transfer to a tertiary facility for further care.

This case is unusual for several reasons. Firstly, it reveals an uncommon life-threatening complication of CPR, hemothorax from a massive bleeding of intercostal arteries leading to hemorrhagic shock and respiratory compromise. Secondly, the PCI received had strict ongoing requirements of antiplatelet therapy which prompted a management dilemma. Lastly, it briefly highlights the challenges of regional hospitals and aims to guide clinicians in the future who may encounter a similar situation and setting. The CARE guidelines were utilized for the reporting of this case [8]. 

## 2. Case Description

A 49-year-old Caucasian man with a previous history of hypertension and Hashimoto’s thyroiditis experienced an out-of-hospital cardiac arrest at his work site, where he received bystander CPR from a colleague. A total of five cycles of CPR and defibrillator shocks were given before he was transported to his local hospital. His electrocardiogram (ECG) showed an anterior ST elevation myocardial infarction (STEMI) with reciprocal changes in his inferior leads. As his local hospital did not have a cardiac catheterization laboratory (CCL), he was loaded with 300 mg of oral aspirin, 180 mg of oral ticagrelor, 40 mg of intravenous (IV) Enoxaparin, 100 mg of therapeutic subcutaneous clexane, and 50 mg of IV tenectaplase. Post-lysis, his chest pain persisted and there was little resolution, i.e., <50% change in ST-T segments and no resolution of his symptoms.

He was transported urgently by air to the closest CCL located 240 km away at the main transfer center in the region. The procedure showed a residual 90% proximal left anterior descending (LAD) artery stenosis, heparin was used during the procedure, and a successful rescue PCI was completed. He was later placed on routine post-STEMI care and a dual antiplatelet (DAPT) regime of 100 mg of oral aspirin and 90 mg of ticagrelor. The post-PCI ECG showed resolution of >50% of his ST-T changes, with resultant T wave inversion in anterolateral leads.

**Figure 1:**
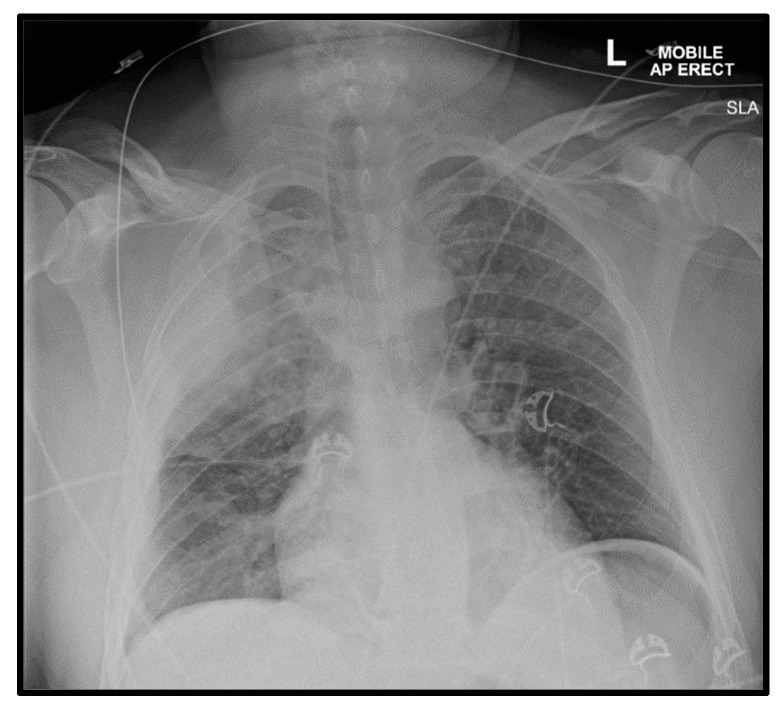
AP erect CXR. Large biconvex right upper chest zone hyperdense lesion. Right lung and left upper lobe contain hazy air space opacity. No pneumothorax. No midline shift. Blunting of right costophrenic angle.

**Figure 2:**
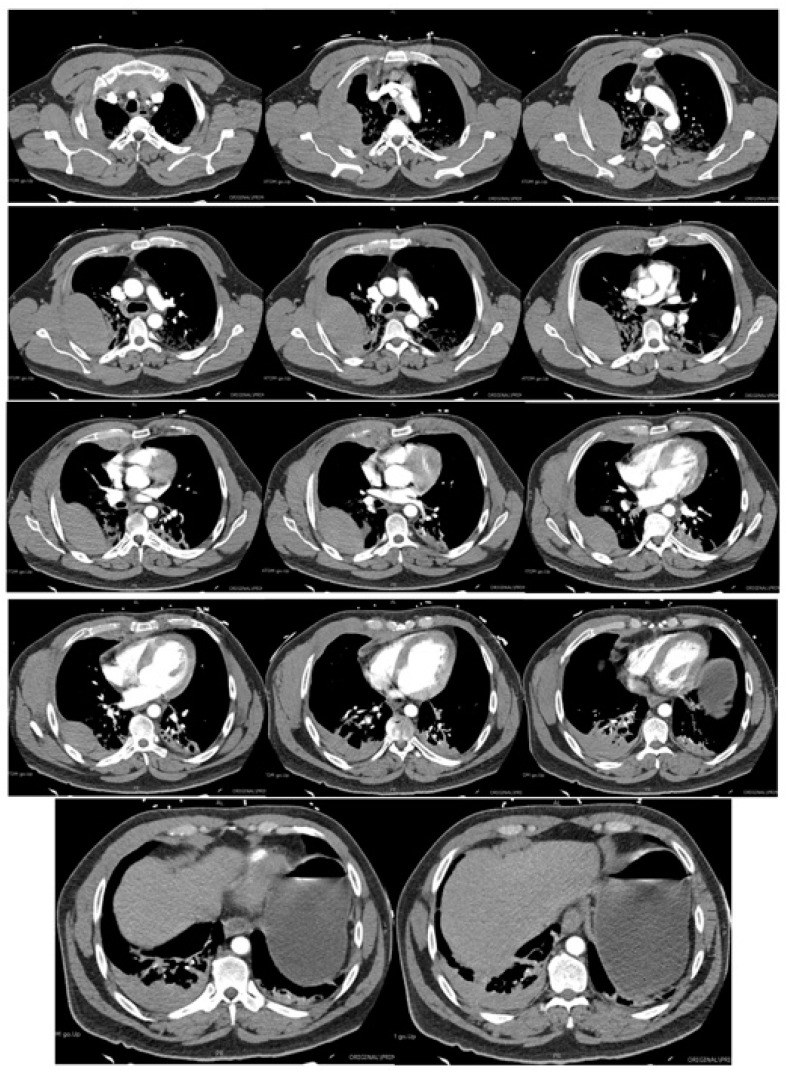
Multiphase CT scan with contrast of chest progressing from superior to inferior. Multiple bilateral rib fractures of 2nd to 6th ribs bilaterally. Moderate right-sided hematoma seen in posterior right extra-pleural space with contrast extravasation seen at the posterior right 3rd and 4th intercostal space suggestive of an active bleed. Right hemothorax. Anterosuperior mediastinal hemoatoma. Collapse consolidation in lung bases. Ground glass opacities with smooth septal thickening in both lung apices. Abdominal organs (not shown) are unremarkable.

Acutely post-procedure, he complained of a right-sided apical pleuritic chest pain. At this time, his Glasgow coma scale was 15, he was afebrile, with a blood pressure of 132/81 mmHg, pulse rate of 72 bpm, tachypneic at 22 respirations per minute with 94% saturation on room air, and he had bilateral crepitations in his middle to lower zones. A chest X-ray (CXR) was performed immediately; the findings and radiograph are shown in Figure 1. A computed tomography (CT) scan with contrast of his chest was performed shortly after; the images and findings are shown in Figure 2. The concern for the hemothorax was increasing given the active bleed, and that the DAPT should not be stopped under any circumstance, due to high risk of stent thrombosis. A general surgical consult was sought which culminated in the recommendation of a cardiothoracic consult. Throughout this period, regular blood was taken to monitor the hemoglobin levels and for a blood group and matching.

The cardiothoracic team phone advice was that he would benefit more from an interventional radiology management perspective and that a chest drain is necessary should signs of respiratory compromise develop. These specialties are not available in the local hospital and required the patient to be transferred to a tertiary hospital approximately 450 km away or an hour by flight. 

**Figure 3:**
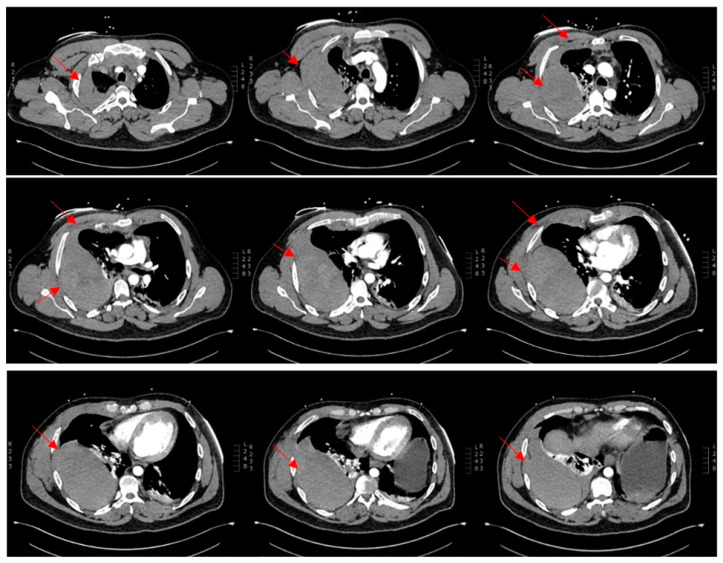
Multiphase CT scan without contrast, arterial and delayed phases, progressing from superior to inferior, compared to previous CT. Increasing size of large right extra-pleural hematoma with multiple sites of active bleeding from intercostal arteries. Moderate-sized right hemothorax seemingly enlarging. Superior mediastinal and right chest wall hematoma.

Arrival at the tertiary hospital happened two hours later, where he started to deteriorate at a faster pace. He remained afebrile, with a blood pressure of 97/49 mmHg, pulse rate of 112 bpm, and tachypneic at 26 respirations per minute with 92% saturation on 15 L of oxygen from a non-rebreather mask. The shock index was 1.2 at this time. A quick point-of-care venous blood gas showed a high anion gap metabolic acidosis with a raised lactate of 3.5. At this point, he entered a hemorrhagic shock along with a respiratory compromise secondary to the expanding hemothorax causing an external restrictive effect on his lung parenchyma. He was quickly sent for a repeat CT chest study without contrast (Figure 3) and was given one unit of packed red blood cells along with 2 g of fibrinogen concentrate. Shortly after, he was being sedated for a selective angiography of his right intercostal arteries and embolization of areas of bleeding.

**Figure 4:**
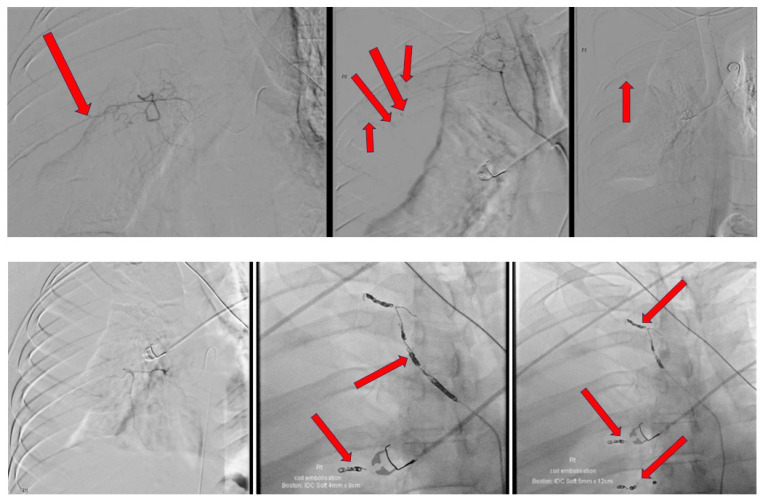
Selective angiography of the right intercostal arteries and embolization of multiple areas of active bleeding with right femoral artery approach—active bleeding from several posterior intercostal arteries with significant right hemothorax. Right hemothorax progressed in size throughout duration of procedure, associated with mediastinal displacement to the left. Contrast extravasation noted from right third and fourth intercostal arteries laterally. Right first, second, third, and fourth posterior intercostal arteries have multiple areas of active bleeding. Particle embolization was performed using Spongostan particles to right first, second, third, fourth, sixth, and seventh intercostal arteries. Multiple detachable coils (measuring 2 mm to 5 mm in diameter) deployed within the bleeding intercostal arteries proximally.

**Figure 5:**
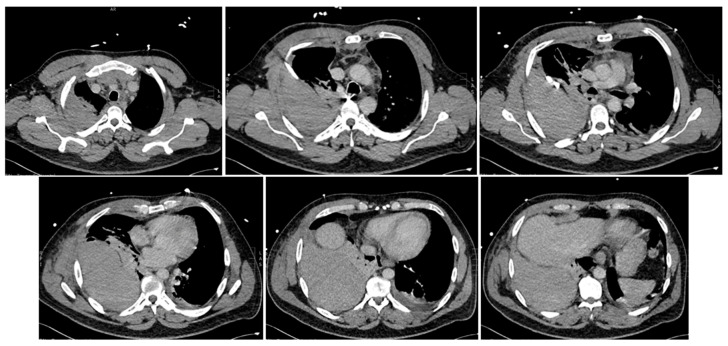
Repeat CT with non-contrast of chest, progressing from superior to inferior. Right intrapleural hematoma increased in size relative to previous findings. New small left pleural effusion. Interstitial and alveolar pulmonary edema. Right lower lobe shows complete collapse. Moderate compressive atelectasis on right upper and middle lobe. Superior mediastinum shows mild hemorrhage.

**Figure 6:**
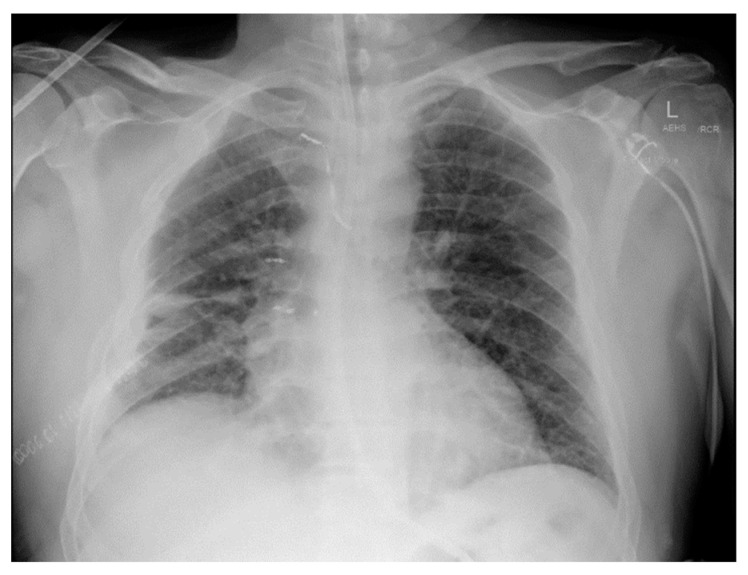
Discharge CXR—nasogastric tube in situ. Persistent bands of atelectasis in right lower zone.

**Figure 7:**
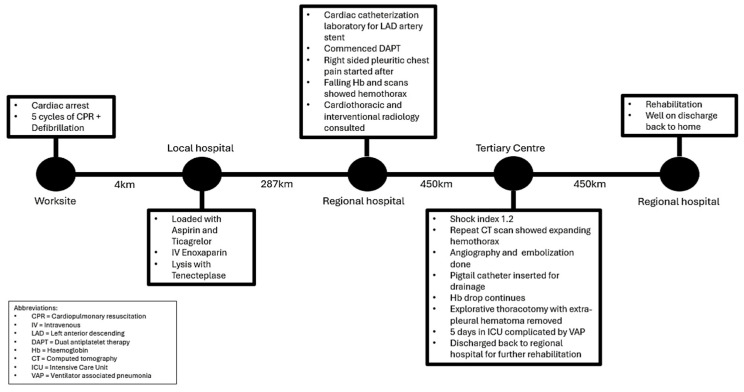
Case timeline of major events at each location, with the distance between each site listed.

The intra-procedural description and images are provided in Figure 4. A 14-French Pigtail intercostal catheter was inserted post-procedure which drained an approximate amount of 700 mL of blood in an hour. The intercostal catheter was left in situ at a draining pressure of negative 20 mmHg. Repeat bloods showed a further significant drop in hemoglobin, from 135 g/dL to 95 g/dL, to 90 g/dL, to 81 g/dL, down to 78 g/dL, with a raised lactate of 3.9 mmol/L. A repeat CT angiogram was carried out to evaluate for any causes of active bleeding; the images can be found in Figure 5. As a result of no significant findings on the CT angiogram to account for the falling hemoglobin, an explorative thoracotomy was agreed upon. An extrapleural hematoma of approximately 2 L of blood was found in the extrapleural space. No active bleeding source was found. There was a collapsed right lung sparing the apical and anterior segments of the upper zone. The right lung fully expanded post-drainage of the blood in the extrapleural space. He remained intubated for 5 days post-explorative thoracotomy on a noradrenaline infusion. Despite developing ventilator-associated pneumonia and being severely deconditioned secondary to these events, he recovered well over the course of a week and was discharged back to the regional hospital for further rehabilitation. CXR prior to discharge is shown in Figure 6. A concise timeline of the key sequences of events at each site is shown in Figure 7. 

## 3. Discussion

High-quality external chest compressions are the cornerstone of quality CPR. It is often recommended to be performed in a “push hard, push fast, do not stop” technique, which enhances survival rate, but inevitably increases the risk of iatrogenic injuries [3,9]. In some instances, this can be accompanied by common injuries such as rib and sternal fractures, specifically, anterior arc of ribs and two-thirds of the sternal body [10,11]. Albeit rare, vital internal organs, including the heart and vessels, can also be affected during CPR [1,10]. Hemothorax can originate from chest wall, intercostal vasculature, internal mammary arteries, mediastinum, and myocardium. However, interestingly, there has been no study that investigated the correlation of duration of CPR and CPR-related pleural complications such as hemothorax [11,12].

In the case presented here, a male experienced right-sided chest pain after receiving PCI to his LAD artery in a regional center after having an out-of-hospital cardiac arrest for which he received five cycles of CPR. An acutely expanding hemothorax occurred shortly after which warranted a prompt referral to a tertiary center. Subsequent selective angiography and embolization of his right intercostal arteries was first carried out by interventional radiologists and an explorative thoracotomy, and later by cardiothoracic surgeons. Ultimately, the patient managed to receive the care he needed promptly at the tertiary center and had an optimal recovery. 

This case aims to objectify the many challenges of a regional hospital, especially in dealing with complex cases as such. The unavailability of cardiothoracic surgery or interventional radiology in our local facility could have unfavorably altered the outcome.

Based on the acuity of the onset, the rapidly expanding hemothorax could be attributed to a combination of the lysis protocol, IV enoxaparin, PCI procedural heparin, and ongoing DAPT use. For the same reasons, the risks of hemodynamic instability outweighed the benefits of performing a tube thoracostomy at the regional center and was thus reserved only if respiratory compromise does develop. The initial normalization of his hemoglobin levels post-PCI, albeit reassuring, was later found to be attributed to a tamponade type effect of the vessels from the CT-proven expanding hemothorax. The limitation of the regional center stops at tube thoracostomies, ROTEM analysis, blood investigations, and blood product transfusion in this context, which is fortunately still lifesaving in many cases.

His further deterioration of the falling hemoglobin after receiving embolization and coiling of the intercostal arteries could possibly be accounted for by the insertion of the intercostal catheter draining at −20 mmHg. Anatomically, the intercostal arteries can be divided into anterior, arising from the internal thoracic artery (also known as the internal mammary artery), and the posterior intercostal arteries, that originate mainly from the thoracic aorta—all of which are high-pressure arterial vasculature [13]. Acutely upon drainage, the tamponade effect likely dissipated, causing a rapid change in the intrapleural pressure leading to the clots, formed with assistance of Spongostan particles used during the interventional radiological procedure, to be forcefully broken down. The presence of intrapleural hematoma later found on explorative thoracotomy further supports this.

Early detection and evaluation of patients with CPR-related pleural complications is essential for effective intervention. The severity of a hemothorax is classified according to the amount of blood within the pleural cavity, with minimal hemothorax being less than 400 mL, medium hemothorax being 400 mL to 1000 mL, and amounts greater than 1000 mL considered to be a massive hemothorax, as in the case of this patient [12]. Thus, appropriate volume evaluation and fluid resuscitation is critical if shock is present. Bedside ultrasound is a non-invasive imaging examination that aids in the assessment and diagnosis of critical patients [14]. Upright CXR and chest CT scans with contrast are also recommended modalities and should be performed in this order [11,12]. However, in this case, only the CXR and chest CT with contrast were performed for the definitive diagnosis. The management goals of a hemothorax should include addressing the source of the bleeding and evacuation of the hematoma [7,12], which could have been the case for our patient if there was not a timely transfer for more sophisticated specialties.

The acute management available for most regional centers includes percutaneous drainage with tube thoracostomy or thoracocentesis. Other complex management options available for tertiary counterparts include interventional radiology for bleeding source control and cardiothoracic surgery for percutaneous video-assisted thoracic surgery (VATS), as well as open thoracotomy [12,14]. Embolization carried out by interventional radiologists is often the preferred first-line management due to being less invasive and better suited for situations at which a patient is deemed not suitable for surgical approaches. However, if preprocedural planning to account for anatomical variations is inadequate, i.e., not performing an initial angiography, it could lead to failure of embolization due to the presence of collateral vessels [15]. However, VATS has also been historically associated with quicker time to recovery and a low mortality risk; it is also often the preferred procedure, should surgery be required, due to the added benefits of being able to evaluate diaphragmatic, mediastinal, or air leak injuries [16]. In our case, the solution to the critical situation benefited from a timely transfer to a tertiary center for further input from these specialties, given the profound risks involved.

## 4. Conclusions

This case highlights an uncommon life-threatening complication of CPR, massive bleeding from intercostal arteries leading to hemorrhagic shock and respiratory compromise from an expanding hemothorax. This complication required two separate complex interventions prior to stability. Early suspicion and identification of bleeding complications associated with CPR is always crucial. This is especially so in similar situations, where the post-PCI DAPT is not to be stopped at any time. For future cases in regional centers, clinicians should be circumspect in their assessments, perform necessary investigations, provide rapid care, consider involvement of the necessary specialties, and transfer to tertiary counterparts to minimize mortality outcomes.

## Data Availability

The datasets used and/or analyzed during the current study are available from the corresponding author on reasonable request.
